# South Asian Children Have Increased Body Fat in Comparison to White Children at the Same Body Mass Index

**DOI:** 10.3390/children4110102

**Published:** 2017-11-22

**Authors:** Emma L. J. Eyre, Michael J. Duncan, Alan Nevill

**Affiliations:** 1 School of Life Sciences, Coventry University, Coventry CV1 2DS, UK; Michael.Duncan@coventry.ac.uk; 2 Faculty of Education, Health and Wellbeing, Wolverhampton University, Wolverhampton WS1 3BD, UK; A.M.Nevill@wlv.ac.uk

**Keywords:** ethnicity, adiposity, obesity, youth, subcutaneous fat

## Abstract

The ability of body mass index (BMI) to predict excess fat in South Asian children is unknown. This cross-sectional study examines the influence of ethnicity on body fatness in children. Weight status and body fat were determined using BMI, waist circumference (WC), two skinfold sites (SF; triceps and subscapula) and leg-to-leg bioelectrical impedance analysis (BIA; Tanita BF350, Tanita, Tokyo, Japan) in 194 children aged 8.47 ± 0.50 years from Coventry, United Kingdom. Biological maturity was also determined. Analysis of covariance (ANCOVA) identified significant differences between ethnic (*p* < 0.001) and gender groups’ BMI (*p* = 0.026), with a significant covariate for skinfold (*p* < 0.001) and bioelectrical impedance (*p* < 0.001). For a given body fat value, South Asian children and females had a lower BMI value (−1.12 kg/m^2^, *p* < 0.001 and −0.50 kg/m^2^, *p* = 0.026, respectively, when adjusted for SF; −1.56 kg/m^2^, *p* < 0.001 and −0.31 kg/m^2^, *p* = 0.16, respectively, when adjusted for BIA) compared with white children and boys. The prediction model including ethnicity, gender and BIA explained 80.4% of the variance in BMI. Maturation was not found to be a significant covariate (*p* > 0.05). To conclude, the findings suggest that BMI cut-points may need to be lowered in South Asian children, and thus age-by-sex-by-ethnicity specific BMI cut-points are needed in children. Further research examining body composition with health parameters in this population is needed.

## 1. Introduction

Over the last decade, a growing body of evidence has shown a development of metabolic disease in childhood that is associated with ethnicity and obesity [1,2,3]. Children from South Asian (SA) backgrounds show abnormal metabolic profiles at young ages compared to white children [3]. Furthermore, South Asian children (5–7 years) are reported to be more likely to be overweight and obese, with a steeper increasing trend in prevalence than for white children [4]. Body size and body fatness differences exist between white and SA children [3,5], with some evidence of these differences during foetal growth [6].

Currently, the use of age- and gender-specific body mass index (BMI) cut-points is advised as a practical estimate of adiposity in children and young people [7]. As such, International Obesity Task Force (IOTF) [8], World Health Organization (WHO) [9], and UK (UK90) [10] growth charts and their associated cut-offs are available. These guidelines do not make any adjustments for ethnicity, which is concerning given that associations are frequently made between BMI and predicted health risks in children [11]. The use of lower BMI thresholds (23 kg/m^2^ increased risk and 27.5 kg/m^2^ high risk) are recommended for SA adults [7]. Consequently, if the BMI–body fatness relationship differs for SA children, as is the case for SA adults, the magnitude of any weight status change in SA children may be misrepresented. 

It is acknowledged that BMI still continues to be routinely used because of its convenience and ability to indicate excess fat in children with a high BMI [12]. The ability of BMI to predict excess fat in SA children who display lower BMI values is unknown. The lack of supporting evidence for the use of separate BMI cut-points for identifying obesity prevalence and increased metabolic risk in SA children enables the current non-ethnic-specific guidelines to be applied to SA children. Given the increased risk of metabolic disease in SA children, potential differences in body composition, and the suggestion of lower BMI values in ethnic adult groups, the true prevalence of obesity may be under-reported, and the identification for at-risk populations may come too late. Examining BMI and fatness together to understand variations in body composition between SA and white children may identify whether lower cut-points are needed to identify early manifestations in at-risk populations to enable early prevention. Therefore, the objective of the study was to examine the influence of SA ethnicity on BMI and fatness, assessed using waist circumference (WC), skinfold (SF) measurements and bioelectrical impedance analysis (BIA) in children. 

## 2. Materials and Methods

A cross-sectional design was employed, collecting primary data in two separate but complementary samples. Following institutional ethics approval (No. P41839), parental informed consent and child assent, a total of 249 children were recruited using cluster sampling from Coventry, United Kingdom. Cluster sampling is a sampling method whereby the researcher divides the population into separate groups on the basis of shared characteristics, called clusters; random sampling of these clusters then occurs. In this study, sampling of ward- and school-level clusters based on high proportions of ethnic minorities at these wards and schools was used. The specific schools within these wards were then randomly selected to minimise potential selection bias. The eligibility criteria included white and SA children aged 8–9 years living in Coventry, United Kingdom. In accordance with the Information Standards Board, the Department for Education and Skills [13], children were classified as SA if they belonged to Indian, Pakistani or Bangladeshi backgrounds. White children included children from any white British, white Irish or white European backgrounds. A prior calculation indicated a sample size of 164 at 95% power; α set at 0.05 would calculate a large effect size. Of the sample of 249 recruited, data on biological maturity was only obtained on a subsample (*n* = 55). For this reason, a two-stage strategy (data divided into two samples) was employed to enable a more holistic evaluation of the research question under scrutiny. Sample 1 thus consisted of a sample of 194 children (112 white and 82 SA; 77 male and 117 female). Sample 2 consisted of 55 children (33 white and 22 SA; 28 male and 27 female) for whom maturation data was collected. Using the two-stage sampling process enabled the outcomes from sample 1 to be validated in an independent but comparable sample whilst also accounting for any effect of maturation.

### 2.1. Anthropometric Assessments

In all the children, height (m) was measured to the nearest 1 mm using a stadiometer (Leicester Portable Height Measure, Seca Instruments, Hamburg, Germany), and body mass (kg) was measured in light indoor clothing to the nearest 0.1 kg (Tanita BF350, Tanita, Tokyo, Japan). BMI was calculated in kg/m^2^. WC was measured using a non-stretchable tape midway between the 10th rib and superior iliac crest. Percent body fat was assessed using leg-to-leg impedance scales (Tanita BF350). BMI and WC data were converted to standard deviation scores (SDS) on the basis of reference curves for children and young people (LMS Growth; Harlow Printing Limited, Tyna and Wear, UK) [10,14]. Skinfold measurements were also taken from two sites (triceps and subscapula) using SF callipers (Harpenden Instruments Ltd., Baty International, West Sussex, UK). Percent body fat from SF measurements was estimated using equations for children [15]. All assessments were undertaken by a researcher and were conducted within the child’s school environment and in line with prior research on anthropometry in paediatric samples [15,16].

### 2.2. Physical Maturity (Biological Age)

Maturity status was determined in a subsample of 55 children (sample 2) using the Mirwald et al. [16] prediction equation. An additional assessment of sitting height (m) measured to the nearest 1 mm using a stadiometer (Leicester Portable Height Measure) was determined. Leg length was calculated by subtracting the sitting height from stature. Using height, sitting height, leg length, chronological age and their interactions, the age-to-peak-height velocity was predicted using gender-specific multiple regression equations [16].

### 2.3. Statistical Analysis

In order to detect differences by ethnicity and gender group in body composition variables, the dependent variable (DV) was firstly BMI (kg/m^2^ and SDS) whilst the body composition was held constant (i.e., SF, BIA and WC); a two-way (ethnicity-by-gender) analysis of covariance (ANCOVA) was employed. Secondly, percent body fat (i.e., SF and BIA) was the DV and BMI was held constant (kg/m^2^ and SDS). In sample 2, the same statistical analysis was conducted, but biological maturity (i.e., age-to-peak-height velocity) was also added as a covariate. Biological maturity data was not available for sample 1 and thus could not be controlled for. In this way, the findings from sample 1 could be independently verified, and at the same time an assessment of any effect of maturation on the research question being examined could be determined. The level of significance was set at *p* < 0.05, and all analysis were conducted using the statistical package for social sciences (SPSS version 20; IBM, Armonk, NY, USA).

## 3. Results

The sample obtained for analysis in sample 1 included 194 children (112 white and 82 SA; 77 male and 117 female) aged 8.47 ± 0.50 years, with a mean BMI of 17.7 ± 3.2 kg/m^2^. Sample 2 consisted of 55 children (33 white and 22 SA; 28 male and 27 female) aged 8.94 ± 0.9 years, with a mean BMI of 18.3 ± 3.7 kg/m^2^. The main findings show that irrespective of the anthropometric measure used, differences in the association between BMI and body fatness are apparent between ethnic groups. The following sections outline the results for each of the comparisons made. 

### 3.1. Sample 1: Dependent Variable, Percent Body Fat

**Figure 1:**
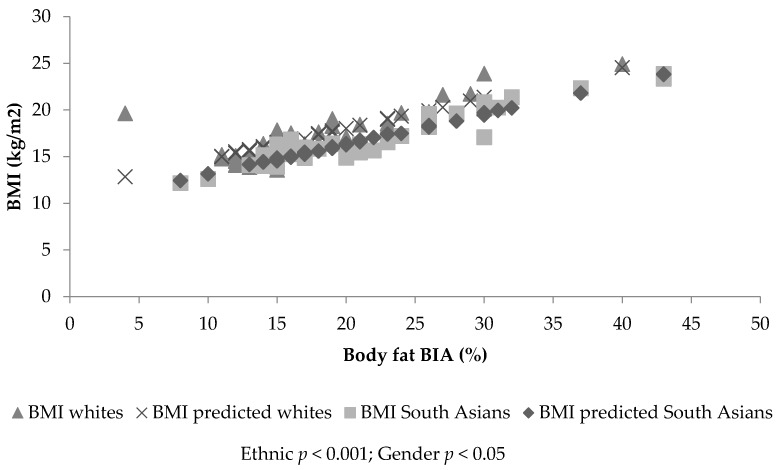
The association between body mass index (BMI) and bioelectrical impedance analysis (BIA) estimated body fat (%), together with the fitted lines for white and South Asian males.

**Figure 2:**
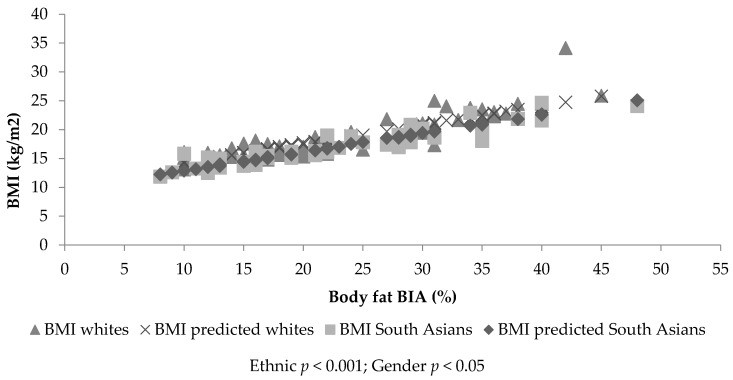
The association between BMI and BIA estimated body fat (%), together with the fitted lines for white and South Asian females.

Figure 1 and Figure 2 identify a linear trend between body fatness and BMI, but show different slopes depending on ethnicity and gender. ANCOVA identified significant percent body fat differences between ethnic groups (SF, *p* > 0.001; BIA, *p* < 0.001) and gender (SF, *p* < 0.001; BIA, *p* = 0.10), with a significant covariate, BMI (SF: β = 1.89, standard error (SE) = 0.072, *p* < 0.001; BIA: β = 2.44, SE = 0.09, *p* < 0.01). Therefore, for a given BMI, SA children and females had significantly increased percent body fat (SF: 2.45%, *p* < 0.001 and 1.70%, *p* < 0.01, respectively—Figure 1 and Figure 2—and BIA: 4.23%, *p* < 0.001 and 1.53%, *p* = 0.01, respectively) compared to white children and boys. The prediction model including ethnicity, gender and BMI explained 80.1% of variance in SF percent body fat and 80.6% in BIA percent body fat. The analysis was re-run with BMI SDS as the covariate; the findings remained the same (SF: β = 4.66, SE = 0.183; BIA: β = 6.86, SE = 0.244). The model including ethnicity, gender and BMI SDS explained 79% of variance in SF percent body fat and 77% of variance in BIA percent body fat. Gender (*p* = 0.035) and ethnic differences (*p* = 0.016) were still found when the sum of SFs was used instead of SF percent body fat and BMI (kg/m^2^) being controlled for (β = 2.531, SE = 0.105). This model explained 82% of the variance in the sum of SFs. No significant ethnic differences were found for WC or ethnicity by gender interactions for any of the assessments of body fat (BF) (*p* > 0.05).

### 3.2. Sample 1: Dependent Variable Body Mass Index

ANCOVA identified significant differences between ethnic (*p* < 0.001) and gender groups’ BMI (*p* = 0.026), with a significant covariate (SF: β = 0.417, SE = 0.016, *p* < 0.001; BIA: β = 0.325, SE = 0.012, *p* < 0.001). Therefore, for a given body fat value, SA children and females have a lower BMI value (−1.12 kg/m^2^, *p* < 0.001 and −0.50 kg/m^2^, *p* = 0.026, respectively, adjusted for SF; −1.56 kg/m^2^, *p* < 0.001 and −0.31 kg/m^2^, *p* = 0.16, respectively, adjusted for BIA) compared with white children and boys (see Figure 1 and Figure 2). This suggests the BMI value was lowered in SA children by −1.12 kg/m^2^ when assessed using SF, and by −1.56 kg/m^2^ when assessed using BIA to identify the same level of BF between white and SA children. The prediction model including ethnicity, gender and SF explained 79.4% of the variance in BMI. The prediction model including ethnicity, gender and BIA explained 80.4% of the variance in BMI. The statistical analysis was re-run replacing BMI (kg/m^2^) with BMI SDS; the findings remained the same. Ethnic and gender differences (*p* < 0.005) in BMI SDS were found, with a significant covariate (SF: β = 0.166, SE = 0.007, *p* < 0.001; BIA: β = 0.128, SE = 0.005, *p* < 0.001). The model including ethnicity, gender and SF percent body fat explained 78% of the variance in BMI SDS. The prediction model including ethnicity, gender and BIA percent body fat explained 76% of the variance in BMI SDS. Ethnic (*p* < 0.05) but not gender differences (*p* > 0.05) were found in BMI SDS and BMI (kg/m^2^) when SF percent body fat was replaced by the sum of SFs (BMI (kg/m^2^): β = 0.319, SE = 0.013; BMI SDS: β = 0.118, SE = 0.006). The model including ethnicity, gender and the sum of SFs explained 82% of the variance in BMI (kg/m^2^) and 75% of the variance in BMI SDS. No significant ethnic differences in BMI were found when WC was used as covariate (*p* = 0.518, *R^2^* = 0.84%). No significance was found for ethnicity by gender interactions (*p* > 0.05).

### 3.3. Sample 2: Dependent Variable, Percent Body Fat

ANCOVA identified significant percent body fat differences between gender (*p* = 0.01) and ethnicity (*p* < 0.001), when BMI (kg/m^2^; *p* < 0.001, β = 2.231, SE = 0.127) was controlled. For a given BMI, girls and SA children had higher percent body fat scores (gender mean difference of 3.33% and ethnic mean difference of 3.87%, respectively). The prediction model including ethnicity, gender and BMI (kg/m^2^) explained 88% of the variance in percent body fat. No interactions were found (*p* = 0.658). When the age-to-peak-height velocity was added to the model, the variables were found not to be a significant covariate (*p* > 0.05). 

When BMI (kg/m^2^) was replaced by BMI SDS as a covariate, ethnic and gender differences were still found in percent body fat (*p* < 0.001) with BMI SDS as a significant covariate (*p* < 0.01, β = 5.948, SE = 1.650). Girls and SA children had higher percent body fat than boys and white children (gender mean difference of 4.22% and ethnic mean difference of 4.74%, respectively). The model including gender and ethnicity explained 85% of the variance. The age-to-peak-height velocity was not a significant covariate, and no interactions between ethnicity and gender were found (*p* > 0.05). 

When WC (cm) was added to the model as a covariate, it was found to be a significant covariate (*p* < 0.01, β = 0.964, SE = 0.064). Ethnic (*p* < 0.001, mean difference of 4.346%) and gender differences (*p* < 0.00, mean difference of 4.698%) were found. The model explained 85% of the variance in percent body fat. When WC SDS was added as a covariate instead of WC (cm), it was found to be a significant covariate (*p* < 0.001, β = 6.171, SE = 0.483), and this model explained 80% of the variance in percent body fat. The age-to-peak-height velocity was not a significant covariate, and no interactions between ethnicity and gender were found (*p* > 0.05).

### 3.4. Sample 2: Dependent Variable Body Mass Index

ANCOVA identified significant BMI (kg/m^2^) differences between gender (*p* = 0.013) and ethnicity (*p* = 0.002), when percent body fat (BIA; *p* < 0.001, β = 0.386, SE = 0.022) was controlled. For a given percent body fat value (BIA), girls and South Asian children had a lower BMI (kg/m^2^) value (gender mean difference of 1.05 kg/m^2^ and ethnic mean difference of 1.32 kg/m^2^, respectively, adjusted for percent body fat). This suggested the BMI value was lowered in South Asian children by −1.32 (kg/m^2^) when assessed using BIA to identify the same level of BF between white and SA children. The prediction model including ethnicity, gender and BMI (kg/m^2^) explained 86% of the variance in percent body fat. No interaction was found (*p* = 0.658). With the age-to-peak-height velocity, the variables were found not to be a significant covariate (*p* > 0.05). 

Gender (*p* = 0.05, mean difference of 0.496) and ethnic differences (*p* = 0.001, mean difference of 0.597) were still found in BMI SDS when percent body fat (BIA) was controlled as a significant covariate (*p* < 0.001, β = 0.138, SE = 0.009). The model explained 82% of the variance in BMI SDS. When the age-to-peak-height velocity was added to the model, this was found to be a significant covariate (*p* = 0.020, β = −0.430, SE = 0.179) in addition percent body fat (*p* < 0.001, β = 0.124, SE = 0.010). Gender (*p* = 0.001, mean difference of 1.187) and ethnic (*p* = 0.002, mean difference of 0.527) differences were found in BMI SDS. This suggested the BMI SDS value was lowered in SA children by −0.527 when assessed using BIA to identify the same level of BF between white and SA children. The complete model explained 84% of the variance. No interaction effects of ethnicity or gender were found. 

WC (cm) was a significant covariate (*p* < 0.001, β = 0.422, SE = 0.496). Gender (*p* = 0.049; girls higher by 0.638) differences were found, but no ethnic differences were found (*p* > 0.05). The model explained 91% of the variance in BMI (kg/m^2^). WC SDS was also a significant covariate (*p* < 0.001, β = 2.525, SE = 0.211). However, when this replaced WC SDS, no ethnic or gender differences (*p* > 0.05) were found. This model explained 74% of the variance. The age-to-peak-height velocity was not a significant covariate in any of these models, and no interactions were found. 

WC (cm) was a significant covariate in BMI (SDS; *p* < 0.00, β = 0.976, SE = 0.064); no ethnic or gender differences (*p* > 0.05) were found. The model explained 82% of the variance in BMI. When WC (cm) was replaced by WC (SDS; *p* < 0.001, β = 0.122, SE = 0.012) and the age-to-peak-height velocity (*p* = 0.003, β = −0.617, SE = 0.198), these were found to be significant covariates. Gender (*p* = 0.18, mean difference of 0.989) but not ethnic differences (*p* = 0.995) were found for BMI SDS. The model explained 78% of the variance in BMI SDS. 

## 4. Discussion

The findings identify ethnic and gender differences in BMI values at the same percent body fat. Specifically, the findings show that SA children and girls have decreased BMI at the same percent body fat compared to matched white children and boys. These findings remained regardless of how the analysis was undertaken (e.g., sum of SFs instead of percent body fat and BMI SDS). Thus, current BMI cut-offs may not be sensitive enough to identify this increased risk in an already at-increased-risk population. The findings suggest that BMI reference curves, such as the British 1990 [8], in children may need to be amended across the gender and age spectrum to consider ethnicity. These findings are in agreement with prior research in older children, suggesting that BMI values should be adjusted by 0.7 to 0.9 kg/m^2^ in 9–10-year-old SA children [10]. Specifically, whilst speculative, this study suggests that at the age of 8–9 years, the cut-points for BMI may need to be lowered in SA children by −1.12 and −1.32 to −1.56 kg/m^2^ (for SF and BIA, respectively). It is not possible to provide this value as the SDS because the age–sex interpretation for this ethnic group will contain error. Given the cross-sectional nature of this study, further research is needed to confirm such suggestions and to provide the age-to-sex-to-ethnicity specific values. The results presented here have both clinical and practical significance. This is because, as they are currently used, BMI cut-points for overweight and obese children may erroneously classify SA children as normal weight when in fact they are overweight. This subsequently results in children who are at an elevated health risk being considered as not at risk. Providing guidance to clinicians and public health professionals on adjusting BMI cut-points for SA children may therefore help in decision-making regarding triggering action for children who may/may not be at elevated health risk, on the basis of BMI. It is also important to note that, although statistically significant, the adjustment might be considered small. However, over a 1 kg/m^2^ difference in the classification of normal-weight and overweight SA children is comparable to data with adult samples that has clinical significance in terms of an increased risk of type 2 diabetes and cardio-metabolic abnormality [7]. Further research needs to examine this further. 

The mechanisms for these ethnic differences may include a combination of lifestyle (i.e., cultural and behavioural) as well as genetic causes. From a genetic perspective, there is some evidence that biological programming during foetal life alters birth size and results in structural and functional changes (e.g., the homeostatic mechanism), which may predispose diseases such as obesity and diabetes [17]. Research by Yajnik et al. [6] supports the notion of biological programming by evidencing that Indian babies are born lighter with more preserved fat mass and a tendency for truncal and central fatness during intrauterine development. The findings from this study identified a similar construct in that SA children aged 8–9 years were lighter but had increased fat, despite being born in the United Kingdom. Currently, there is no evidence that tracks SA children from the neonatal period to adulthood; such information would provide valuable insight into SA children’s body composition with growth and maturation. 

Furthermore, the evolutionary role of a thrifty genotype is also argued. It is suggested that this genotype would enable fat storage during times of plentiful food and fat, acting as a useful survival mechanism during disasters, feast and famines [18] and as a key feature of the country of origin. Ethnic groups then face challenges in lifestyle, such as a plentiful constant supply of food and decreased physical activity associated with their immigration experience [19]. This increase in supply may result in increased fat storage, coupled with the adoption of a less-active lifestyle in westernised worlds [20,21]. While a plausible mechanism, given that the children were born in the United Kingdom, it is likely that other lifestyle factors may also play a role in the development of adiposity.

From a lifestyle perspective, physical activity is negatively associated with body fat in SA children [22]. Several studies have reported the low adherence by SA children to physical activity guidelines when compared to white children [23,24]. Furthermore, an experimental study has identified that percent body fat can be decreased in SA children by increasing physical activity [25]. The role of cooking practices is underexplored in SA children, and thus this may also prove to be important in the development of fat [26].

Despite the use of cluster sampling, self-selection may have provided a bias in the study. The use of ethnic-specific algorithms for SA children when estimating body fat was not available, and thus universal algorithms were used, which may have underestimated or overestimated fat. However, when the sum of SFs was used instead of the percent body fat, the same findings were found. An understanding of maturational differences across ethnic groups is limited, but maturation may have an effect on body composition, thus explaining these differences. However, in the current study, when maturation was considered, it was not significant as a covariate. This suggests that maturation may not have a meaningful association in relation to the ethnic differences reported here. This is likely to be influenced by the pre-pubertal nature of the children. Additionally, this may have been influenced by the assessment of maturation obtained for this study, which was predictive in nature and has not been extensively examined in an ethnic population. A physical examination would provide the most accurate assessment of biological maturity, but this is an invasive assessment, particularly for field-based assessments. Age-to-peak-height velocity assessments provide a valid, non-intrusive and simple method for predicting maturity in children [27]; in this study, maturation did not change the outcomes. Further research needs to confirm the effects of maturation assessed using a physical examination across the childhood process in relation to growth changes between ethnic groups. Additionally, WC was found to be a significant covariate of BMI but not percent body fat, and ethnic and gender differences no longer remained when WC was controlled. It is speculated that this may be a result of the children being pre-pubertal and thus geometrically similar [28]. Furthermore, there are several limitations of BMI, including its accuracy, which varies by the degree of body fatness, and thus BMI is sensitive to distinguishing between fat mass and lean mass. This is further complicated by natural growth changes across childhood years [12]. This can be particularly challenging when making comparisons between groupings and with clinical outcomes, particularly given that cut-points based on clinical outcomes would need longitudinal assessments conducted over a span of several decades. While BMI may not present the most effective way of assessing fatness, it is still a widely used screening tool and is routinely assessed at reception- and year 6-levels as part of the national child measurement programme in the United Kingdom. For this reason, guidance is needed on the basis of these assessments and how they may differ by ethnic groupings to trigger action. Our study identified the importance of obtaining combined measures, for example, body fat and measures of girth or stature, to fully understand body composition differences in pre-pubertal children and across ethnic groupings. 

In conclusion, the study identified that SA children had higher body fat at a lower BMI value than age-matched white children, irrespective of biological age. This may suggest that the current age- and sex-specific BMI cut-points may misclassify SA children’s body composition.
